# Policy making to address imbalances in human resources for eye health in rural Kenya

**Published:** 2018-07-31

**Authors:** Michael Mbee Gichangi, Hazel Miseda Mumbo

**Affiliations:** 1 Head: Ophthalmic Services Unit: Ministry of Health, Nairobi, Kenya.; 2 Managing Director: Mizel Consultants Company Ltd, Nairobi, Kenya.

A human resources mapping study in 2011 highlighted that Kenya had attained the WHO target of 4 ophthalmologists per million population. However, 49% of the ophthalmologists were based in the capital city and served only 8% of the overall population. The cataract surgical rate was 553 operations per million population per year, which is significantly below the required rate of 2,000 per million population per year.

Shortages in the health workforce in Kenya are aggravated by the country's limited training capacity and the steady, internal migration of health workers from rural to urban areas, which is driven by economic, social, professional and security factors.^1^ This is an example of the inverse care law,^2^ which states that medical services are inverse to the need in the population.

This problem called for a comprehensive and integrated investment in incentives to recruit and retain personnel in rural areas.^3^,^4^

We conducted a literature review, collected data from existing policy documents, and carried out interviews with key informants on strategies for the recruitment and retention of (eye) health workers in Kenya. We wanted to collect evidence about:

- Trends in the recruitment and retention of health workers in rural districts
- Existing policies, strategies and interventions to retain health workers, and their impact
- Existing retention incentive schemes, and their impact
- Lessons learned and guidelines for non-financial incentive packages to promote the retention of health workers.

The evidence was discussed in 2018 at the annual Kenya Health Forum meeting,^5^ and the following actions (supported by changes in policy) were agreed.

- Setting up an integrated human resource information system to plan the training and distribution of the workforce
- Planning national, comprehensive training needs assessments (TNAs) to gather detailed evidence about which eye care professionals are needed where. This takes place every two years, and the the next one is planed for 2018
- Developing and implementing a human resource advisory group responsible for improving the welfare of the workforce in order to improve productivity and retention, especially in rural areas.

The Ophthalmic Services Unit of the Kenyan Ministry of Health now works with partners to offer affordable scholarships as an incentive for people from marginalised rural counties wanting to join the eye health workforce.

The progress of these initiatives will be evaluated at the Kenya Health Forum meeting in 2019.
